# TLR-mediated stimulation of APC: Distinct cytokine responses of B cells and dendritic cells

**DOI:** 10.1002/eji.200636483

**Published:** 2007-11

**Authors:** Tom A Barr, Sheila Brown, Gemma Ryan, Jiexin Zhao, David Gray

**Affiliations:** Institute of Immunology and Infection Research, School of Biological Science, University of EdinburghEdinburgh, UK

**Keywords:** B cells, Cytokines, Dendritic cells

## Abstract

In addition to their role in humoral immunity, B lymphocytes are important antigen-presenting cells (APC). In the same way as other APC, B cells make cytokines upon activation and have the potential to modulate T cell responses. In this study, we investigated which mouse B cell subsets are the most potent cytokine producers, and examined the role of Toll-like receptors (TLR) in the control of secretion of IL-6, IL-10, IL-12 and IFN-γ by B cells. Production of some cytokines was restricted to particular subsets. Marginal zone and B1 cells were the predominant source of B cell IL-10 in the spleen. Conversely, follicular B cells were found to express IFN-γ mRNA directly *ex vivo*. The nature of the activating stimulus dramatically influenced the cytokine made by B cells. Thus, in response to combined TLR stimulation, or *via* phorbol esters, IFN-γ was secreted. IL-10 was elicited by T-dependent activation or stimulation through TLR2, 4 or 9. This pattern of cytokine expression contrasts with that elicited from dendritic cells. QRT-PCR array data indicate that this may be due to differential expression of TLR signalling molecules, effectors and adaptors. Our data highlight the potentially unique nature of immune modulation when B cells act as APC.

## Introduction

It is now generally accepted that recognition of foreign organisms or damaging circumstances by cells of the innate immune system, *i.e.* antigen-presenting cells (APC), triggers immune response initiation. Such recognition events lead to maturation of these APC to enable presentation of processed antigen to T cells in immunogenic form, involving provision of costimulation. Innate cells recognise pathogen-associated molecular patterns (PAMP) [1, 2] or tissue damage [3–5] by their expression of pattern recognition receptors (PRR). One of the most important groups of PRR is the Toll-like receptor (TLR) family of type I transmembrane proteins, characterised by highly divergent, leucine-rich extracellular domains and highly conserved Toll-IL-1R (TIR) cytoplasmic domains. To date, 12 murine and human TLR have been described. Humans express TLR1–10 and 13 and the mouse expresses TLR1–9, 11 and 13 (TLR11 is also called TLR12 by Tabeta *et al.* [6]). The ligands for TLR1–9 and 11 have now been described [7–10]. TLR are linked *via* adapter molecules to intracellular signalling pathways that generally lead to transcription of NF-κB target genes. Among these targets are cytokine genes, and it is these that are crucial not only for the initiation of the adaptive response but also in shaping its direction. For example, in the absence of the major TLR adapter, MyD88, there is little IL-12 production and a failure of the Th1 response [11]. In relation to this process, most attention has centred on dendritic cells (DC) as the main initiating APC. However, B cells are no different from other APC in their need for maturation signals to allow them to attain their full presentation capacity. Furthermore, it is now being revealed that TLR ligation of B cells may also be important for antibody production in normal and autoimmune responses 12–17. To date, there has been no systematic study of TLR expression by B cells and how they respond to various TLR signals.

B cells do not participate significantly as initiators of the immune response, either because they cannot activate naive T cells [18] or because the frequency of antigen-specific B cells, the only efficient B cell presenters, is far too low. However, they do become important APC later in the primary response and in the secondary one, where their increased numbers means they may be the predominant antigen-presenting force [19]. We and others have demonstrated an important contribution of B cells as activators of T cells *in vivo* [20], a contribution that is both early (clonal expansion and Th differentiation; [21, 22]) and late (immune regulation; [22–24]) and is delivered by secretion of cytokines, in particular IL-10 [23, 25, 26]. The B cell compartment comprises several subsets, and it is becoming apparent that not all are equal in their capacity to present antigen to T cells; thus, Attanavich *et al.* [27] report that marginal zone (MZ) B cells are much more active in this respect than follicular (FO) B cells. As well as the MZ *versus* FO B cell dichotomy (identified by CD21/CD23 expression), the B cell compartment is split into B1 and B2 populations. MZ and FO B cells are B2 B cells. B1 B cells largely express the marker CD5 and are found in the peritoneal cavity, and also the spleen. B1 cells respond to T-independent antigens and are responsible for the production of most background serum Ig (mostly IgM); they do not participate significantly in T-dependent responses [28]. B1 and B2 cells seem to arise as separate lineages [29, 30], although the developmental relationship between B1 cells and MZ B cells is still not clear [31].

Published reports suggest that B cells acting as APC during the primary response lead to a Th2-biased response [22, 32], in contrast to DC, which often, as a result of IL-12 production, drive a Th1 response [33]. A full functional, molecular explanation for this distinction has yet to be provided. The maturation of DC into effector DC [34] which have the ability to differentially drive Th responses is influenced by TLR ligation [35–37]. As a parallel, it is likely that TLR ligation on B cells influences their role as APC, and in directing T cell differentiation. We have looked to see if the contrasting roles of B cells and DC as APC can be explained by differences in their expression of TLR or their reactivity to TLR ligands. We find that while mouse B cells express mRNA for all TLR, their response to the triggering of these receptors was clearly distinct.

## Results

### B cell subsets show differential *ex vivo* expression of IL-6, IL-10 and IFN-γ mRNA

**Figure 1:**
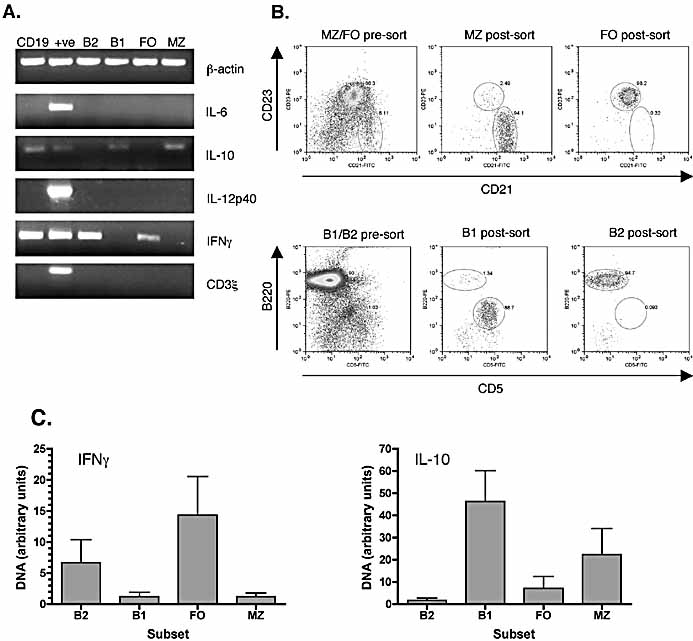
Cytokine mRNA expression by B cells. B cells were purified by CD19^+ve^ MACS, then FACS sorted according to expression of cell surface markers. (A) RT-PCR analysis of mRNA extracted from cell sorts; +ve is the positive control (unsorted splenocytes). Semi-quantitative RT-PCR for β-actin, IL-6, IL-10, IL-12p40 and CD3ξ are shown. (B) Flow cytometry-based sorting of B cell subsets on CD19^+ve^ pre-sorted splenocytes. Upper panels show MZ/FO sorting based on expression of CD21 and CD23. Lower panels show B1/B2 cell sorting based on expression of CD5 and B220. (C) QRT-PCR analysis of mRNA from MZ/FO and B1/B2 cell sorts. cDNA concentrations, relative to the β-actin housekeeping control gene, are indicated for IFN-γ and IL-10. Error bars indicate SEM on PCR performed on two separate RNA samples, each extracted from pooled cells from five mice.

B lymphocytes isolated from non-immunized C57/BL6 mice by CD19^+ve^ MACS sorting were found to express mRNA for IL-10 and IFN-γ as determined by reverse transcription (RT)-PCR (see Fig. 1A). We also performed RT-PCR reactions on B cell mRNA for the detection of IL-4 and IL-5, but no message for these cytokines was detected (data not shown). IL-10 and IFN-γ mRNA could not be attributed to contaminating non-B cells in preparations, as no message for CD3ξ could be detected, indicating an absence of NK and/or T cells (see Fig. 1A). Additionally, only trace contaminants of CD3^+ve^ and CD11c^+ve^ cells were detected in sorts (0.16 and 0.076%, respectively – data not shown). CD5^+ve^ B1 cells from the peritoneal cavity are known to produce IL-10 [38]. To investigate cytokine production by splenic B cell subsets, we separated purified CD19^+ve^ B cells into B1/B2 cells and MZ/FO subsets by FACS. Fig. 1B illustrates high-purity B cell subset sorts. mRNA was extracted from these samples and assayed by RT-PCR for the presence of IL-6, IL-10, IL-12p40 and IFN-γ. As expected, splenic B1 cells expressed IL-10 mRNA, but not mRNA for IFN-γ. B2 cells did not express IL-10 but did express IFN-γ mRNA. This dichotomy was also observed in MZ and FO B cells, in which MZ B cells from non-immunized animals expressed IL-10 mRNA but not IFN-γ message, while FO B cells expressed IFN-γ but not IL-10 mRNA. IL-12p40 mRNA was not detected in the CD19^+ve^ B cell preparation or in any of the enriched B cell subset sorts (Fig. 1A). The lack of detectable IL-10 message in the B2 mRNA was unexpected, as this sort would presumably contain some MZ cells. It seems likely that the number of MZ cells present in the B2 sort was below the detection limit of the IL-10 PCR. The B cell subset cytokine dichotomy was quantified by quantitative real-time reverse transcription (QRT)-PCR analysis of IL-10 and IFN-γ mRNA in B1/B2 MZ/FO subsets (Fig. 1C). FO and B2 cells were found to express high levels of IFN-γ mRNA but negligible levels of IL-10. B1 cells expressed the highest levels of IL-10 mRNA, followed by MZ cells. These subsets expressed negligible levels of IFN-γ mRNA (Fig. 1C). The differences in IFN-γ and IL-10 gene transcript levels point to the potential for B cell subsets to produce different cytokines based on their phenotype.

### Murine B cells express TLR1–9

**Figure 2:**
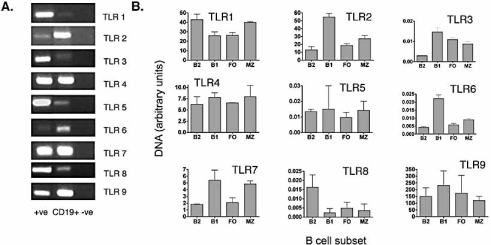
B cell expression of TLR mRNA. (A) RT-PCR performed on mRNA extracted from highly purified CD19^+^ B unsorted splenocytes (+ve) and negative control (H_2_O). (B) Differential expression of TLR mRNA by MZ, FO, B1 and B2 B cell subsets. All values are given as DNA concentrations relative to the expression levels of the β-actin housekeeping gene. Results shown are based on the mean values calculated from two separate reactions performed on pooled cells from five mice per group.

Given the importance of TLR in driving APC maturation, we sought to quantify TLR transcript levels of B cell subsets. mRNA from purified CD19^+ve^ splenic B cells was extracted and analysed by RT-PCR to investigate the TLR repertoire of these cells. CD19^+ve^ B cells were found to express mRNA for all of the nine TLR tested in this investigation (see Fig. 2A).

### MZ and B1 cells express a different TLR repertoire to FO and B2 cells

QRT-PCR analysis of mRNA extracted from B cell subsets confirmed the previous observation that B cells express mRNA for all nine TLR in all subsets tested (Fig. 2B). TLR1, 2, 4, 7 and TLR9 transcripts were high amongst B cells, while comparatively TLR3, 5, 6 and 8 showed low expression. Levels of TLR mRNA differed between B cell subsets. B1 and MZ B cells displayed TLR mRNA expression levels distinct from B2 and FO cells. B1 and MZ cells expressed higher levels of TLR2, 6 and 7 transcripts than their B2 and FO counterparts. These subsets also expressed lower levels of TLR8 mRNA than B2 and FO cells (Fig. 2B).

### Activation of B cells *via* TLR

**Figure 3:**
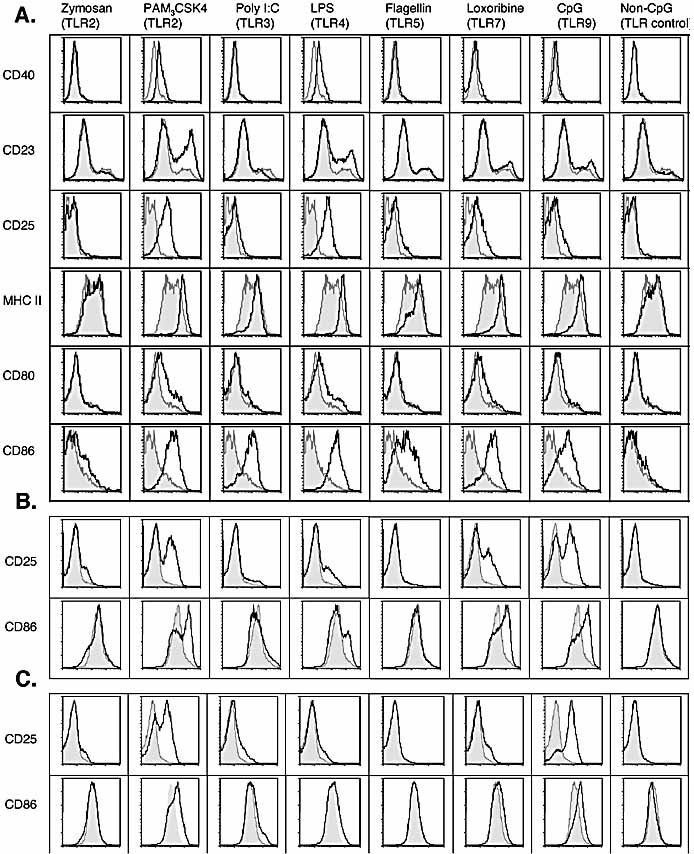
Activation of B cells through TLR stimulation. B cells were cultured overnight with a range of TLR agonists as indicated at the top of the graph. Cells were then stained with mAb against a range of B cell activation markers, as indicated on the left. For each plot, the mean fluorescent intensity is plotted against % of maximum, with the filled grey histogram representing staining for unactivated B cells (incubated overnight in the absence of any TLR stimulus) and the open histogram representing staining of cells activated in the presence of the corresponding TLR agonist. (A) Splenic CD19^+ve^ B cells. (B) MZ B cells (CD19^+ve^, B220^+ve^, CD21^high^, CD23^int/low^). (C) FO B cells (CD19^+ve^, B220^+ve^, CD21^+ve^, CD23^high^). Representative data from three separate experiments are shown.

Activation and subsequent up-regulation of costimulatory molecules by B cells in response to TLR stimulation *in vitro* was measured by flow cytometry following overnight stimulation (Fig. 3A). LPS (TLR4) and PAM_3_CSK4 (TLR2) both up-regulated CD40 expression. These two agonists were also the only stimuli to induce a substantial increase in CD23 expression, although a small increase was apparent in CpG-treated B cells. CD25 expression was substantially enhanced by PAM_3_CSK4 and LPS treatment and to a lesser extent by loxoribine, flagellin and CpG. MHC class II expression was augmented by all of the TLR agonists tested, excluding zymosan and the negative control non-CpG oligonucleotide. CD80 expression levels were slightly increased in B cells stimulated through TLR2, 4, 7 and 9. CD86 expression was strongly up-regulated through stimulation *via* TLR2, but only with PAM_3_CSK4 and not zymosan. Up-regulation of CD86 was also observed following stimulation through TLR3, 4, 5, 7 and 9. Again non-CpG oligonucleotide (TLR negative control) did not induce up-regulation of this costimulatory molecule.

FACS-sorted MZ and FO subsets were also incubated overnight with each of the TLR ligands and assayed for up-regulation of CD25 and CD86 (see Fig. 3B, C). CD25 was up-regulated on MZ cells by stimulation through TLR2, 7, 9 and to a lesser extent through TLR4. Similarly, MZ expression of CD86 was increased by TLR2, 4, 7 and 9 stimulation. FO B cell activation followed a similar pattern to that displayed by whole CD19 B cells and MZ cells; however, their propensity for activation was generally lower (see Fig. 3B). TLR2-and 9-stimulated FO cells showed elevated CD25 and CD86. A small up-regulation of these activation markers was also seen with TLR3, 4 and 7.

### TLR stimulation elicits distinct cytokine secretion patterns from B cells and DC

**Figure 4:**
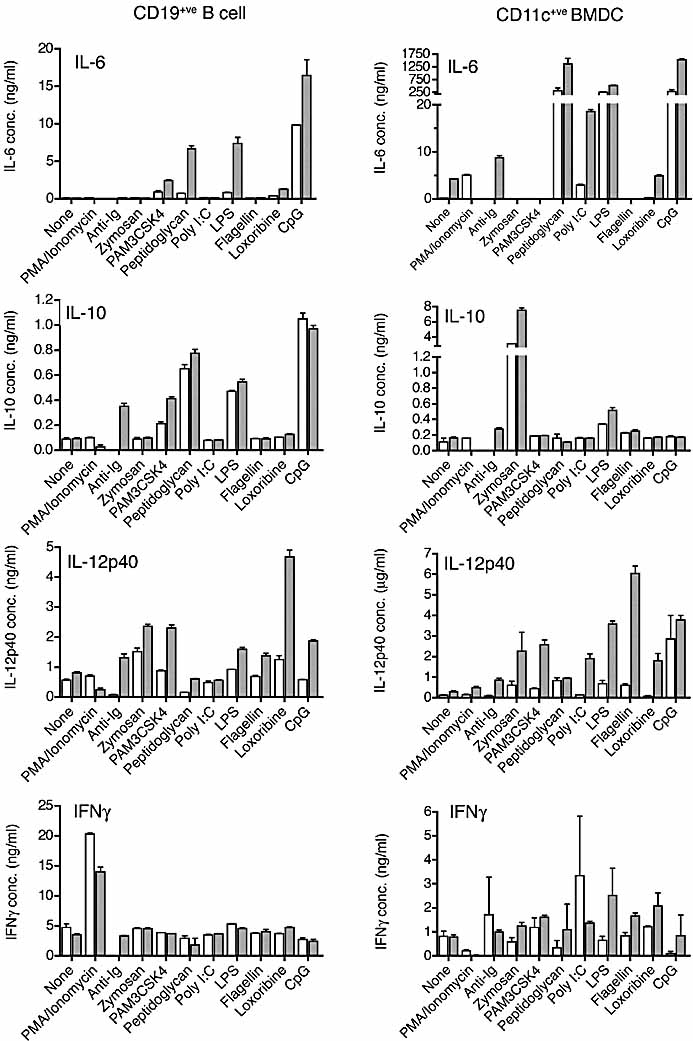
Cytokine production by DC and B cells stimulated with TLR agonists. Highly purified B cells and DC were cultured at 2 × 10^6^ cells/mL in the presence of a range of stimuli, either alone (white bars), or in the presence of anti-CD40 antibody (grey bars). After 5 days, culture supernatants were harvested and assayed by ELISA to determine the concentrations of IL-6, IL-10, IL-12p40 and IFN-γ. Data shown are based upon triplicate cultures of pooled cells sorted from four mice and are representative of four separate experiments. Error bars represent SEM. The limits of detection for each ELISA were as follows: IL-6 = 0.3 ng/mL; IL-10 = 0.1 ng/mL; IL-12p40 = 0.1 ng/mL; and IFN-γ = 0.8 ng/mL.

CD19^+ve^ B cells and CD11c^+ve^ bone marrow-derived DC (BMDC) were cultured with agonists for TLR2, 3, 4, 5, 7 and 9. After 5 days, supernatant was collected to quantify cytokine levels. Time course experiments to quantify cytokine levels at days 1, 3 and 5 were performed, of which day 5 was found to be the peak of cumulative cytokine production (data not shown). B cells and DC responded very differently to TLR stimulation with respect to their cytokine secretion (Fig. 4). CpG (TLR9), LPS (TLR4) and peptidoglycan (PGN – TLR2) were found to be the most potent inducers of B cell-derived IL-6, being especially effective in conjunction with CD40 stimulation. Low levels of IL-6 were also elicited from B cells treated with PAM_3_CSK4, PGN (TLR2) and LPS (TLR4). In contrast, DC secreted IL-6 levels some tenfold higher than those seen in B cells with PGN (TLR2), LPS (TLR4) and CpG (TLR9). Additionally, DC responded to poly I:C (TLR3 in conjunction with CD40 stimulation) and to a lesser extent to CD40 stimulation alone.

B cells activated in a T-dependent fashion, *via* simultaneous ligation of the BCR and CD40, are known to make IL-10, and this observation was confirmed by our data (Fig. 4). Additionally, we found that B cells stimulated with PAM_3_CSK4 (TLR2), PGN (TLR2), LPS (TLR4) or CpG (TLR9) also secreted IL-10. Costimulation with anti-CD40 antibody led to a small increase in IL-10 production through TLR2 and 4, but not with TLR9. This contrasts with the DC response to TLR stimulation, where production of IL-10 was strongly induced by zymosan treatment (TLR2/dectin-1), but not with PAM_3_CSK4 or PGN (TLR2). IL-10 was also elicited from DC treated with LPS. As with B cells, CD40 stimulation of DC synergised with the TLR stimulus to enhance IL-10 production.

IL-12p40 secretion was strongly induced in DC treated with all of the TLR agonists tested, especially PGN (TLR2), LPS (TLR4), flagellin (TLR5) and CpG (TLR9). As expected from previous studies [35, 37], CD40 synergised with TLR stimulation to augment cytokine secretion. Very low levels of IL-12p40 were also detected in some TLR-treated B cell cultures (loxoribine, CpG, and to a lesser extent with zymosan or PAM_3_CSK4). It is noteworthy that IL-12p40 levels detected in B cell cultures were at the threshold of sensitivity for the assay and were 1000-fold less than IL-12p40 elicited from DC in the same conditions.

Finally, IFN-γ secretion from B cells was not detected in response to any single TLR or T-dependent stimulus, only following mitogenic stimulation with PMA and ionomycin or with discrete combinations of TLR agonists (see below). IFN-γ secretion by BMDC in response to TLR stimulation or with PMA and ionomycin was also negligible.

ELISA were also performed for the detection of B cell-derived IL-4, IL-17 and IL-23. Of these cytokines only IL-23 was detected, but in response to PMA and ionomycin and not to any TLR stimulus (data not shown).

### Signalling through TLR4 induces differing gene expression profiles in DC and B cells

The observation that B cells and DC display different cytokine profiles in response to the same TLR stimulus was intriguing and we sought to identify the mechanisms underlying this observation. In order to address this, we looked at the expression levels of a number of TLR signalling-associated genes before and after LPS stimulation using a QRT-PCR-based array system (see Table 1). Several striking differences were observed between the transcript expression levels between the two cell types. In terms of TLR adaptor molecules, DC greatly up-regulated expression of heat shock protein 1A (Hspa1a, 34-fold increase) whereas B cells down-regulated expression (approximately twofold decrease). In contrast, B cells increased expression of Bruton's tyrosine kinase (Btk), whereas DC decreased expression. In terms of the effector molecules Caspase 8 (Casp8), Fas-associated death domain (FADD) and TNFR-associated factor 6 (TRAF6), only FADD was differentially expressed with a fivefold decrease in DC following TLR4 stimulation. Expression of genes involved in the JNK/p38 pathway were more highly expressed in DC than in B cells. In particular, FBJ osteosarcoma oncogene (c-Fos) and Jun oncogene (Junc) showed a small increase in expression in DC, and Elk-1 showed a large decrease in B cells. The most striking differences in expression were seen in prostaglandin-endoperoxide synthase 2 (Ptgs2), C-type lectin domain family member 4 (Clec4e) and Cxcl10 expression, which were all vastly increased in LPS-treated DC, but only moderately so in B cells.

**Table 1:** QRT-PCR analysis of TLR signalling-associated genes in DC and B cells^a^)

	BMDC	CD19^+ve^ B cells
Adaptors		
Btk	0.39	4.86
Hspa1a	34.34	0.44
Myd88	0.78	1.21
Tirap	0.38	0.68
Tollip	1.2	0.64
Effectors		
Casp8	1.07	1
FADD	0.2	0.99
TRAF6	1	0.61
NF-κB pathway		
Ccl2	2.96	0.44
Il-1α	19.11	0.44
NF-κB	1.39	1.16
NF-κB2	1.19	1.16
**JNK/p38 pathway**		
c-Fos	1.78	0.38
Junc	2.05	0.54
Map2K4	0.65	0.95
Elk-1	0.79	0.08
NF/IL-6 pathway		
Il-6R	0.08	0.29
IRF-1	2.33	1.11
IRF-3	0.39	0.41
PTGS2	557.18	0.44
Other		
Clec4e	30.57	1.69
CXCL10	97.07	7.57
Eif2ak2	1.27	3.43

a DC or B cells (2 × 10^6^) were incubated for 24 h with LPS. Following RNA extraction, DNase treatment and cDNA synthesis QRT-PCR analysis was performed using a TLR-focused array. Values in the table indicate fold change in expression level compared with unstimulated cells, whereby less than 1 indicates down-regulated expression.

### Defined combinations of TLR agonists cooperate to elicit IFN-γ production from CD19^+ve^ B cells

**Figure 5:**
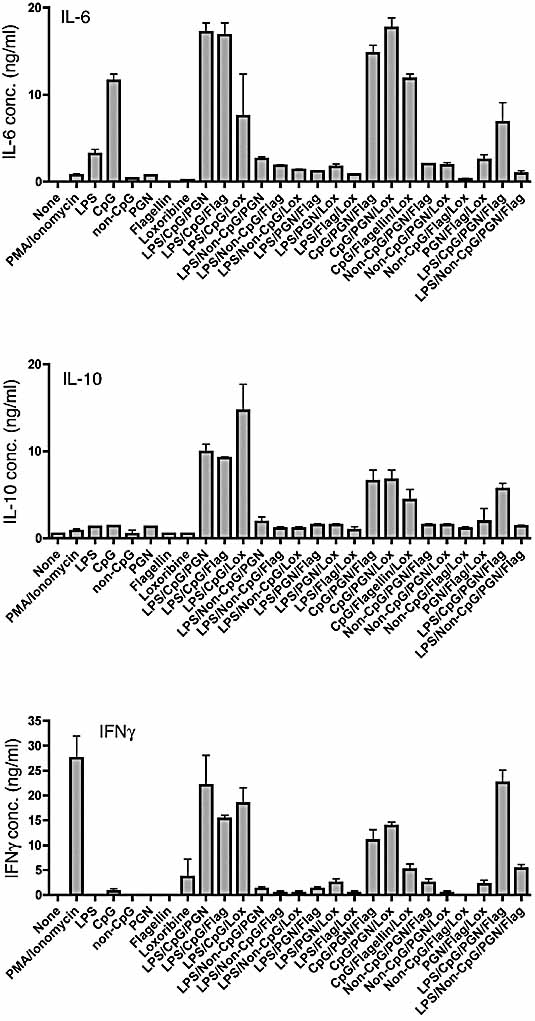
Combined TLR stimulation of B cells. CD19 MACS-purified B cells were cultured with defined combinations of TLR agonists as indicated on the *x* axis at 2 × 10^6^ cells/mL. After 5 days of *in vitro* stimulation, supernatants were assayed for levels of IL-6, IL-10 and IFN-γ. Data shown are based on triplicate cultures of pooled cells from groups of four mice and are representative of four separate experiments. Error bars indicate SEM. Limits of detection were as follows: IL-6 = 0.3 ng/mL; IL-10 = 0.1 ng/mL; and IFN-γ = 0.8 ng/mL.

It is has been previously demonstrated that defined combinations of TLR agonists can synergise in their stimulation of DC to induce increased Th1 polarisation through the provision of increased levels of IL-12 and IL-23 [39]. In order to address the potential of TLR synergy on B cells, we stimulated CD19^+ve^ splenic B cells with combinations of TLR2, 4, 5, 7 and 9 agonists (Fig. 5). Intriguingly, we found that combined stimulation through TLR2, 4 and 9 (with CpG being particularly potent) elicits IFN-γ production by B cells, a cytokine previously only produced in response to phorbol esters. These combinations also led to enhanced IL-10 and IL-6 (see Fig. 5) production from B cells.

### TLR stimulation elicits distinct cytokine secretion patterns from MZ and FO B cells

**Figure 6:**
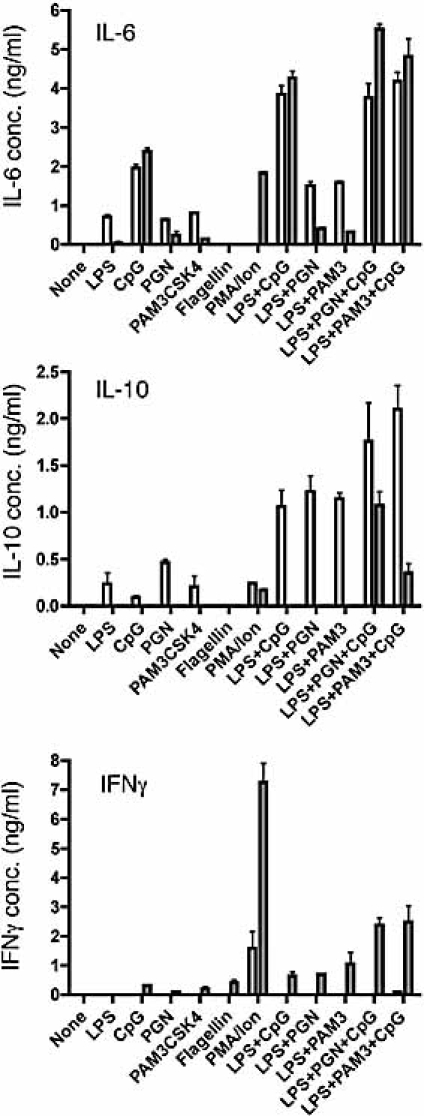
TLR-mediated cytokine production by purified B cell subsets. B cells isolated by a combination of CD19 MACS selection and FACS were cultured at 1 × 10^6^ cells/mL with stimuli known to induce cytokine secretion by whole CD19^+ve^ B cells, as indicated on the *x* axes. After 5 days in culture, supernatants were harvested and assayed to determine concentrations of IL-6, IL-10 and IFN-γ by ELISA. For each graph, MZ B cells are represented by white bars and FO B cells by grey bars. Data presented are based on duplicate cultures of cells pooled from four mice and representative of four separate experiments. Error bars represent SEM. Limits of detection were as follows: IL-6 = 0.3 ng/mL; IL-10 = 0.1 ng/mL; and IFN-γ = 0.8 ng/mL.

Highly purified MZ and FO B cell subsets were stimulated with TLR agonists and combinations that had previously been demonstrated to elicit IL-10, IL-6 and of IFN-γ from whole CD19^+ve^ B cell stimulations (Fig. 6). We found that MZ B cells were the main source of IL-10 from splenic B cell populations; they secreted IL-10 in response to PAM_3_CSK4 and PGN (TLR2), LPS (TLR4) and CpG (TLR9) whereas FO B cells did not. Additive or synergistic effects were seen with TLR2, 4, 9 stimulation of MZ B cells, with maximal production from stimulation through all three receptors *via* LPS, PAM_3_CSK4 (or PGN) and CpG. This triple stimulus also induced a small amount of IL-10 production by the FO cells, suggesting that, rather than a complete inability to produce IL-10, the threshold for production from these cells is significantly higher.

Conversely, the only splenic subset capable of IFN-γ secretion after TLR stimulation were FO B cells. Low levels were detectable in most FO cultures, but combined stimulation through TLR2, 4 and 9 or *via* mitogenic stimulation *via* phorbol esters elicited high levels of production. As with MZ-derived IL-10 production, IFN-γ elicited from FO B cells was maximal in triple stimulation through TLR2, 4 and 9. Low levels of IFN-γ could be elicited from MZ B cells, but only with PMA/ionomycin treatment.

Finally, in contrast to IL-10/IFN-γ polarisation, IL-6 did not appear to be preferentially secreted by either subset. Both MZ and FO B cells secreted IL-6 under several conditions, including LPS, CpG, PMA/ionomycin and combinations containing LPS or CpG. However, the MZ subset provides IL-6 more readily in response to LPS stimulation. CpG appeared to be a particularly potent driver of IL-6 production by both subsets of B cells. Both TLR2 ligands tested (PGN and PAM_3_CSK4) were equivalent in their induction of IL-6, IL-10 and IFN-γ.

## Discussion

The main findings of this study are that mouse B cells can express all TLR (TLR1–9) and that these function to activate the cell in a variety of ways. However, this broad TLR expression is only true of unseparated (CD19^+ve^) splenic B cells, as subsets of B cells exhibit a differential TLR expression. Of the TLR ligands tested, only those binding to TLR2, TLR4 and TLR9 elicited significant cytokine secretion from B cells (IL-6 and IL-10) when used alone. However, stimulation through TLR2, 4 and 9 in concert elicited IFN-γ and enhanced levels of IL-10 and IL-6. When compared to TLR-induced cytokine secretion by DC, B cells exhibited a distinct pattern, producing IL-6 and IL-10 in response to TLR4 ligation, which gave IL-6 and IL-12 in DC. The probable mechanism underlying divergent cytokine production in response to TLR stimulation appears to be the differential use of TLR-associated signalling pathways, as array-based analysis of these cell types reveals distinct gene profiles in the two cell types in response to the same TLR stimulation. Additionally, cytokine production is also influenced by the type of responding B cell; MZ cells produce IL-6 and IL-10 whereas FO B cells secrete IFN-γ and IL-6.

The expression and function of TLR1–9 on mouse B cells highlights the importance of PAMP recognition by B cells. By analogy with DC, it seems likely that TLR ligation is an important maturation signal for B cell APC function. This is demonstrated by the up-regulation of MHC class II and the costimulation molecules, CD80 and CD86, by all the TLR ligands with the exception of zymosan. This is also true of the sorted MZ and FO subsets, although clearly MZ cells showed a higher propensity for activation. Clearly, the activated phenotype is not entirely indicative of cytokine effector function. For example, the TLR7 ligand, loxoribine, up-regulates CD25 and CD86 in a similar way to LPS or CpG, yet despite this, no IL-10 or IFN-γ is elicited. Thus, the activation of the B cell is required but is not necessarily indicative of cytokine production. It may be possible that those TLR ligands inducing activation may elicit other cytokine responses not covered in this study (although IL-4, IL-17 and IL-23 were also tested with negative results; data not shown) or perhaps only act in a fully stimulatory manner in the presence of other costimulatory molecules absent in a B cell-only culture. It is interesting that CD40, a known T-dependent regulator of costimulatory function of APC, is only up-regulated on B cells stimulated with TLR2 or TLR4 ligands. This may imply that additional T cell help is required for optimal responses in these settings, a hypothesis born out by the observation that cytokine production is enhanced with costimulation by anti-CD40 antibody. Not all B cell subsets express all the TLR. Compared to other subsets, FO and B2 B cells express less mRNA for TLR2, 6 and 7 and roughly equivalent amounts of TLR1, 4, 5 and 9 mRNA. Interestingly, MZ B cells and B1 cells show a clear similarity in TLR expression, displaying increased levels of TLR2, 6 and 7, along with decreased levels of TLR8. Both of these B cell subsets made IL-10 when stimulated with PAM_3_CSK4 or PGN, indicating that TLR2 seems functionally active in this setting. We do not know, at present, the significance of increased levels of TLR6 and 7, and decreased levels of TLR8. TLR2 and TLR6 are known to form a heterodimeric receptor utilised in the recognition of bacterial PGN [40], so the similarity in expression levels is not unexpected. In some subsets mRNA expression was found to be very low for some certain TLR. Whether this level of expression is functionally relevant remains to be determined.

It is striking that not all TLR2 ligands stimulated B cells; thus, stimulation with PAM_3_CSK4 or PGN, but not with zymosan, elicited IL-10 secretion. The likely reason for this is that B cells do not express dectin-1 [41], which acts together with TLR2 as a β-glucan receptor [42]. Lack of dectin-1 expression could provide an explanation for why B cells cannot respond to zymosan, while they can respond to PAM_3_CSK4, as recognition of the latter is mediated by TLR2 alone. DC, which do express dectin-1, responded to zymosan by secreting IL-10. Interestingly, however, they do not do so in response to PAM_3_CSK4, indicating that TLR2 itself is wired differently in B cells and DC. Recently, Syk kinase has been shown to be necessary for the zymosan-elicited IL-10 response by DC [43]. However, as Syk was shown to associate with the cytoplasmic tail of dectin-1, this again suggests that the TLR2-mediated IL-10 response in B cells is elicited *via* a different signalling pathway. The cytokine response to PGN is comparable in B cell subset stimulations (Fig. 6.), indicating that it is indeed the dectin-1 pathway which is significant in DC. The response to the other TLR2 ligand, PGN, in this study is more complicated. As this molecule is isolated from bacteria, it is often contaminated with LPS. Indeed DC produce IL-6 in response to PGN, whereas they do not with PAM_3_CSK4, which could well indicate an LPS response (although the lack of IL-10 or IL-12 in these cultures suggests that any contamination may be at low levels). Our data illustrate an important distinction between murine and human B cell TLR repertoires. We show that murine B cells express TLR1–9, while a recent study of human tonsillar B cells showed them to express TLR1, 4, 6, 7, 8, 9 and 10, but not TLR2 [44]. This contrasts starkly with the results here, in which we show expression of TLR2 mRNA and show that B cells respond to TLR2 ligands in a variety of ways, most importantly by IL-10 and IL-6 production.

In order to investigate the dichotomy in the B cell and DC responses to TLR stimulation, we undertook a QRT-PCR array-based analysis of TLR-associated signalling molecules and adaptors. The differences in transcript levels between B cells and DC in these assays were striking, with a number of TLR signalling-associated molecules showing differential expression. For example, Btk expression is highly up-regulated in B cells following TLR4 stimulation, whereas in DC expression is reduced. Several reports have recently implicated Btk as an essential molecule in LPS signalling (see [45] for a review). Much work on Btk has focused on its role as a regulator of B cell activation, maturation and development through BCR-mediated activation; however, the finding that B cells up-regulate Btk transcription upon LPS stimulation is intriguing, particularly in light of the fact that DC do not do likewise. Clearly, this differential alone is unlikely to account for the differences seen between B cells and DC in their responses to TLR. Indeed, from our data, it is clear that a number of molecules are involved including, but almost certainly not limited to, Btk, CCL2, FADD, IL-1α, Ptsg2, Clec4e and CXCL10. Thus, it appears that B cells and DC may respond by provision of different cytokines to the same TLR ligand through differential use of intracellular signalling pathways and their associated adaptor molecules. It will be intriguing to see how other cell types and other signalling pathways are exploited in this way as this is at the core of innate responses; namely, how pleiotropic events are elicited from conserved PAMP receptors.

One of the consequences of differential TLR expression by MZ and FO B cell subsets is a dichotomy in their ability to make cytokines. Thus, MZ B cells make IL-10 (in response to TLR2 and 4), while FO B cells do not, but do make IFN-γ. The dichotomy is not absolute as with very strong stimulation (PMA/ionomycin) purified MZ cells did secrete low levels of IFN-γ. Conversely, when stimulated through TLR2, 4 and 9, FO cells produced a little IL-10. Both MZ and FO B cells make IL-6, and CpG stimulation seems an important driver for this response. MZ B cells represent an important source of IL-10, directly *ex vivo*, following TLR2-and 4-mediated stimulation and after T-dependent activation (*via* CD40). In a similar way to DC [35, 37], we found that B cells exhibited an additive cytokine response when exposed to both TLR and CD40 signals. Based on the data presented, we would predict that *in vivo* IL-10 production would be optimal only after TLR-activated B cells interact with CD154-expressing CD4^+^ T cells. As mentioned already, it is intriguing that the two TLR that drive strong IL-10 responses also up-regulate CD40, which in turn augments the IL-10 response.

B1 cells have been known for over a decade to make IL-10 [38]. Our finding that MZ cells share the capacity to produce IL-10 highlights another similarity between these B cell subsets. The relative IL-10 productivity of B1 cells and MZ B cell populations *in vivo* is not clear. Although there are more MZ B cells in the spleen, the greater capacity of B1 cells to secrete IL-10 suggests that the two populations contribute similarly to the splenic IL-10 response.

What function does IL-10 made by these B cells have in the immune response? IL-10 is a potent inhibitor of inflammatory responses that exerts part of its effect by the suppression of IL-12 transcription and secretion by DC [46]. In a recent study by Sun *et al.* [47], IL-10 produced by CD5^+ve^ B cells in response to CpG stimulation was shown to down-modulate the pro-inflammatory response of DC. Clearly, the IL-10 elicited by TLR2 and 4 activation of B cells could be acting in a similar fashion. Although we stated that splenic B1 and MZ B cell populations may secrete similar amounts of IL-10 *in vitro*, it seems likely that, *in vivo*, MZ B cells are better placed to influence DC (and hence T cell) differentiation, as there are DC within the MZ that migrate to T cell zones upon TLR4 ligation [48, 49]. The systemic immunosuppressive role of B cell-derived IL-10 *in vivo* has been demonstrated previously by us [23], and by other laboratories [25, 50], although whether this effect is exerted directly on DC or *via* other cell populations remains uncertain. An interesting possibility is that the IL-10 made by B cells is utilised in the generation of inducible populations of regulatory T cells, some of which (Tr1 cells) are known to be IL-10 dependent in their development [51].

Previous work has shown that CpG acts directly on B cells, resulting in activation and antibody secretion [52]. Krieg and colleagues investigated human B cell responses to TLR stimulation and showed that human B cells responded *via* TLR9 to secrete IL-10 and that there was synergy if the BCR was also ligated [53]. Similarly, Sun *et al.* [47] have recently shown that CD5^+ve^ B cells stimulated with CpG secrete IL-10. Also, in a mouse model of systemic lupus erythematosus (SLE), MZ B cells have been shown to respond to CpG by secretion of IL-10 [54, 55]. Our data agree that MZ B cells can act as a significant source of IL-10, and that one way of achieving this response is through TLR9 stimulation *via* CpG.

Another function of IL-10 is to promote B cell growth, differentiation and antibody class switching [56–58]; so its production by B cells could have an autocrine effect [59]. IL-10 secretion from B cells has been demonstrated in human B cell lymphomas [59]. Thus, in response to TLR and T-dependent activation signals, B cell-derived IL-10 may act to promote the ensuing response by expanding the B cell population and inducing class switching to appropriate isotypes.

Although IFN-γ secretion by B cells has been reported previously [60], our demonstration that this cytokine can be secreted by B cells in response to defined TLR combinations is an exciting and novel finding. We found that combined stimulation through TLR2, 4 and 9 with TLR ligands (PAM_3_CSK4 or PGN, LPS and CpG) induced IFN-γ secretion by B cells, which was not apparent when any of these stimuli were used alone, in conjunction with BCR signalling or with costimulation through CD40. This is an important consideration when assessing the physiological importance of *in vitro* TLR stimulations. The situation *in vivo* during a bacterial infection would certainly provide a range of TLR signals to the B cell. For instance, B cells from *Salmonella*-infected mice make very significant amounts of IFN-γ when restimulated with bacterial antigens (Mastroeni, Barr and Gray, unpublished). This B cell-derived IFN-γ may act on T cells or even, in an autocrine fashion, on B cells themselves. Thus, it may regulate homing of immature B cells [61] and has been shown to be important during *Borrelia burgdorferi* infection [62]. As APC, IFN-γ-secreting B cells could influence Th1 differentiation and, in addition, initiate isotype switching (to IgG2a/c and IgG3). Autocrine IFN-γ may be particularly important as a switch factor for IgG3 in response to T-independent antigens [63]. Interestingly, a recent report by Ehlers *et al.* [13] demonstrated defective class switching to IgG2a in MyD88-deficient animals, although the possibility that this was an autocrine B cell effect was not investigated.

Most important of all, our results show that there are distinct response patterns from different APC populations stimulated *via* the same TLR. Thus, B cells make IL-10 and IL-6 following TLR4 and 9 ligation, in contrast to DC that make IL-12, IL-6 and IL-10. In addition, IL-6 is elicited in B cells *via* TLR2, 4 and 9, but by TLR3, 4 and 9 in DC. This suggests that the composition of the membrane-proximal TLR signalling elements (adaptors) differs between these two important APC populations. The reasons for this differential APC response are surely bound up in the different *in vivo* APC function of DC and B cells. We would suggest that soon after the first wave of DC-mediated T cell activation/differentiation is underway, both the TLR-mediated and adaptive activation of B cells gives rise to cells that are capable of modulating the dominant T cells response, either to down-regulate the response in general, to prevent strongly polarised responses [23–25] or in some cases (IFN-γ) to reinforce particularly important responses.

## Materials and methods

### Mice

Mice were bred and maintained in specific pathogen-free conditions at the School of Biological Sciences Animal Facility at the University of Edinburgh. All experiments utilised non-immunised female C57BL/6 mice at 6–10 wk of age. Experiments were covered by a Project Licence granted by the Home Office under the Animal (Scientific Procedures) Act 1986. This licence was approved locally by the University of Edinburgh Ethical Review Committee.

### Preparation and sorting of highly purified B cells

Splenic B cells were isolated from naive female C57BL/6 mice using standard magnetic sorting techniques. Following manual disruption in complete medium [Iscove's modied Dulbecco's medium (IMDM) + 5% FCS and penicillin/streptomycin] and lysis of red blood cells, splenocytes were labelled with anti-CD19 microbeads, washed and sorted over two consecutive LS columns in accordance with the manufacturer's instructions (Miltenyi Biotech, Bisley, UK). Passing cells over a second column led to purities in excess of 99% CD19^+ve^ cells (data not shown). These highly purified B cells were analysed by FACS for contaminating T cells and DC using anti-CD3 and anti-CD11c antibodies. B cells were further purified by flow cytometric sorting carried out following the CD19 enrichment described above. FO B cells were defined as CD19^+ve^, CD21^high^ and CD23^high^. MZ B cells were defined as CD19^+ve^, CD21^high^ and CD23^med/low^ (see Fig. 1A). B1 cells were defined as CD19^+ve^, B220^int^ and CD5^int^, whereas B2 cells were defined as CD19^+ve^, B220^high^ and CD5^–ve^ (see Fig. 1B). FO, MZ and B2 subset sorts were routinely >94% pure. B1 cell sorts were routinely >85% pure.

### Preparation of BMDC

BMDC were prepared using a modified version of the protocol originally described by Inaba *et al.* [64]. Briefly, marrow was harvested from the femurs and tibias of female C57BL/6 mice. Following red blood cell lysis, cells were seeded at 3.75 × 10^5^ cells/mL in RPMI 1640 supplemented with 10% FCS and 5% supernatant from GM-CSF transfected X-63 cells [22. After 3 days, the incubation medium was aspirated and fresh GM-CSF-supplemented medium added. This washing process was repeated again at day 6. Cells were harvested at day 7, giving approximately 90% pure CD11c^+ve^ and MHC class II^+ve^ cells. These cells were then labelled with anti-CD11c microbeads and positively sorted using standard MACS protocols. This regime yielded greater than 97% pure BMDC (data not shown).

### Nucleic acid extraction and PCR analyses

mRNA was extracted from cells using the Micro-FastTrack 2.0 mRNA extraction kit (Invitrogen, Renfrew, UK) in accordance with the manufacturer's instructions. Contaminating DNA was removed from samples by DNase treatment with Ambion's DNA-Free (Ambion Europe, Dublin, Ireland) prior to second-strand synthesis. cDNA was generated from mRNA by reverse transcription using the StrataScript second-strand synthesis kit (Stratagene, UK). Semi-quantitative RT-and QRT-PCR analysis was carried out using a range of primers, the sequences, melting temperatures and product sizes of which are given in Table 2. Where possible, primers were designed to span exon junctions to further reduce the possibility of amplification of non-specific products from contaminating genomic DNA. PCR products were visualised on ethidium bromide-labelled 1.8% agarose gels using standard techniques. QRT-PCR was performed using Roche SYBR green and the LightCycler thermal cycler (Roche Diagnostics, Lewes, UK). Products were quantified by comparing expression levels of the gene of interest relative to the expression levels of the housekeeping control gene β-actin.

**Table 2:** Primer sequences used for RT-PCR and QRT-PCR analysis in this study^a^)

Gene	Primer	Sequence	Amplicon size (bp)	Tm
**β-Actin**	B-act FOR	TGG AAT CCT GTG GCA TCC ATG AAA C	**348**	**58.0**
	B-act REV	TAA AAC GCA GCT CAG TAA CAG TCC G		**58.0**
**GAPDH**	GAP FOR	TTC ACC ACC ATG GAG AAG GC	**236**	**59.4**
	GAP REV	GGC ATG GAC TGT GGT CAT GA		**59.4**
**CD3ξ**	CD3e FOR	CCT TTT CTC CTC ATC CTC CC	**250**	**54.0**
	CD3e REV	TGC ACT CCT GCT GAA TTT TG		**50.0**
**IL-6**	IL6 FOR	CCT CTC TGC AAG AGA CTT CCA TC	**520**	**68.3**
	IL6 REV	AGC CAC TCC TTC TGT GAC TCC AG		**70.9**
**IL-10**	IL10 FOR	GGT TGC CAA GCC TTA TCG GA	**190**	**59.4**
	IL10 REV	ACC TGC TCC ACT GCC TTG CT		**61.4**
**IL-12p40**	IL12 FOR	GGA AGC ACG GCA GCA GAA TA	**180**	**59.4**
	IL12 REV	AAC TTG AGG GAG AAG TAG GAA TGG		**61.0**
**IFNg**	IFNg FOR	AGC GCT GAC TGA ACT CAG ATT GTA G	**243**	**58.0**
	IFNg REV	GTC ACA GTT TTC AGC TGT ATA GGG		**56.0**
**TLR1**	TLR1 FOR	GGA TGT GTC CGT CAG CAC TA	**340**	**59.4**
	TLR1 REV	TGT AAC TTT GGG GGA AGC TG		**57.3**
	QRTTLR1-1	TAC AGT TCC TGG GGTTGAGC	**216**	**54.0**
	QRTTLR1-2	TAG TGC TGA CGG ACA CAT CC		**54.0**
**TLR2**	TLR2 FOR	CAG ACG TAG TGA GCG AGC TG	**390**	**61.4**
	TLR2 REV	GGC ATC GGA TGA AAA GTG TT		**55.3**
	QRTTLR2-1	CGT TGT TCC CTG TGT TGC T	**119**	**51.0**
	QRTTLR2-2	AAA GTG GTT GTC GCC TGC T		**51.0**
**TLR3**	TLR3 FOR	GAG GGC TGG AGG ATC TCT TT	**353**	**59.4**
	TLR3 REV	TGC CTC AAT AGC TTG CTG AA		**55.3**
	QRTTLR3-1	TTG CGT TGC GAA GTG AAG	**406**	**48.0**
	QRTTLR3-2	TAA AAA GAG CGA GGG GAC AG		**52.0**
**TLR4**	TLR4 FOR	GCT TTC ACC TCT GCC TTC AC	**361**	**59.4**
	TLR4 REV	CGA GGC TTT TCC ATC CAA TA		**55.3**
	QRTTLR4-1	TTC ACC TCT GCC TTC ACT ACA	**225**	**52.0**
	QRTTLR4-2	GGG ACTT CTC AAC CTT CTC AA		**52.0**
**TLR5**	TLR5 FOR	GCT TTG TTT TCT TCG CTT CG	**342**	**55.3**
	TLR5 REV	ACA CCA GCT TCT GGA TGG TC		**59.4**
	QRTTLR5-1	CAG GAT GTT GGC TGG TTT CT	**169**	**52.0**
	QRTTLR5-2	CGG ATA AAG CGT GGA GAG TT		**52.0**
**TLR6**	TLR6 FOR	GCA ACA TGA GCC AAG ACA GA	**349**	**57.3**
	TLR6 REV	GTT TTG CAA CCG ATT GTG TG		**55.3**
	QRTTLR6-1	ATG GCA CAG CGG ACT TAC TT	**170**	**52.0**
	QRTTLR6-2	ATG AGA GCC CAG GTT GAC AG		**54.0**
**TLR7**	TLR7 FOR	ATT CAG AGG CTC CTG GAT GA	**264**	**57.3**
	TLR7 REV	AGG GAT GTC CTA GGT GGT GA		**59.4**
	QRTTLR7-1	GCT GTG TGG TTT GTC TGG TG	**270**	**54.0**
	QRTTLR7-2	CCC CTT TAT CTT TGC TTT CC		**50.0**
**TLR8**	TLR8 FOR	TCC TGG GGA TCA AAA ATC AA	**302**	**53.2**
	TLR8 REV	AAG GTG GTA GCG CAG TTC AT		**57.3**
	QRTTLR8-1	GAC TTC ATC CAC ATC CCA AA	**156**	**50.0**
	QRTTLR8-2	TCC CAA TCC CTC TCC TCT AA		**52.0**
**TLR9**	TLR9 FOR	ACC CTG GTG TGG AAC ATC AT	**341**	**57.3**
	TLR9 REV	GTT GGA CAG GTG GAC GAA GT		**59.4**
	QRTTLR9-1	GAA AGC ATC AAC CAC ACC AA	**304**	**50.0**
	QRTTLR1-2	ACA AGT CCA CAA AGC GAA GG		**52.0**

a Nucleotide sequences for each of the RT-PCR and QRT-PCR products (excluding those in the signalling array analysis) are listed, including indications of PCR product size and theoretical melting temperature (Tm).

### QRT-PCR array analysis

An RT^2^ Profiler PCR array system was used to quantify gene expression using a TLR signalling-focused array in compliance with the manufacturer's instructions (SuperArray Bioscience Corporation, Frederick, MD). Briefly, after 24 h of stimulation of CD19^+ve^ B cells or BMDC with LPS at 1 µg/mL, total RNA was extracted from 2 × 10^6^ cells using the Qiagen RNeasy minikit and DNase (Qiagen, Crawley, UK) treated. Reverse transcription was carried out using the RT^2^ PCR array first-strand kit (SuperArray). Quality control of all samples was carried out on a SuperArray QC array. PCR were performed using the Chromo4 real-time PCR system, and data were analysed by the ΔΔC_t_ method using data analysis templates from SuperArray.

### *In vitro* TLR stimulation assays

Highly purified CD19^+ve^ B cells, CD11c^+ve^ BMDC and B cell subsets were cultured at 2 × 10^6^ cells/mL in complete medium under a range of conditions used to mimic various modes of B cell activation. T-dependent activation was mimicked by simultaneous stimulation through the BCR and CD40 using agonistic antibodies against the Ig kappa light chain (clone 187.1) and anti-CD40 antibody (clone FGK-45), which were produced in-house by standard mAb purification techniques on hybridoma supernatants. These mAb were used at 15 and 10 µg/mL, respectively. Endotoxin-free TLR ligands were purchased from InVivogen (Autogen Bioclear UK Ltd.) and added to cultures, either alone or in the presence of anti-CD40 antibody, at concentrations detailed below. TLR2 agonists zymosan, PGN and PAM_3_CSK4 were used at 10, 10 and 0.2 µg/mL, respectively; poly I:C (TLR3 ligand) was used at 25 µg/mL; LPS (TLR4 ligand) from *E. coli* 0:111 B4 was used at 1 µg/mL; flagellin (TLR5 ligand) was used at 0.1 µg/mL; loxoribine (TLR7) was used at 100 µM, and the TLR9 ligand CpG (ODN 1826) and its non-CpG-containing oligonucleotide control were used at 25 µg/mL. CpG and non-CpG sequences were as follows: ODN 1826 5′-TCC ATG ACG TTC CTG ACG TT-3′ and ODN 1826 control 5′-TCC ATG AGC TTC CTG AGC TT-3′. PMA and ionomycin (Sigma, UK) were used at 50 ng/mL and 1 µg/mL, respectively. Cells were cultured under these conditions for 1–5 days. After 1 day, an aliquot of cells was removed for FACS analysis of activation markers. At days 1, 3 and 5, aliquots of supernatant were removed for ELISA assay of cytokine concentrations.

### Cytokine ELISA

Supernatants from TLR-stimulated cultures were assayed to determine cytokine concentrations by standard capture ELISA techniques using commercially available paired antibody sets for IL-6, IL-10, IL-12p40 and IFN-γ (BD Pharmingen, San Diego, CA). Briefly, Nunc Maxisorp plates (Fisher Scientific, Loughborough, UK) were coated overnight at 4°C with capture mAb at 5 µg/mL in PBS. Plates were then blocked for 1 h at room temperature with 3% BSA. Supernatants were then added to the plate and left for 2 h at room temperature. Following washing, biotinylated capture mAb was applied and the plates incubated for a further hour at room temperature. Streptavidin-alkaline phosphatase was then added and, following incubation at room temperature and washing, *p-*NPP substrate was added. Plates were read at 405 nm once the substrate had developed, and cytokine concentration was determined by extrapolation from the standard curve.

### Flow cytometric analysis

Following overnight incubation with the various TLR agonists, B cells were assayed for the expression of various activation markers. The antibodies used, all obtained from BD Pharmingen, were as follows; anti-CD23-PE, anti-CD25-APC, anti-CD80-PE, anti-CD86-PE, anti-MHC class II-FITC, and anti-CD40-biotin. With each of the relevant mAb in FACS buffer (PBS + 1% BSA + 0.05% NaN_3_), 5 × 10^5^ cells were incubated for 20 min on ice. Cells were then washed three times and analysed on a FACSCalibur flow cytometer (BD Pharmingen). Data were analysed using FlowJo software (Tree Star Inc.).

## Abbreviations:

BMDC: bone marrow-derived dendritic cell
fo: follicular
MZ: marginal zone
PAMP: pathogen-associated molecular pattern
PGN: peptidoglycan
QRT: quantitative real-time reverse transcription
